# The Netrin-4/Laminin γ1/Neogenin-1 complex mediates migration in SK-N-SH neuroblastoma cells

**DOI:** 10.1080/19336918.2018.1506652

**Published:** 2018-08-30

**Authors:** Andrea A. Villanueva, Sofía Puvogel, Pablo Lois, Ernesto Muñoz-Palma, Manuel Ramírez Orellana, Fabiana Lubieniecki, Fernando Casco Claro, Iván Gallegos, Javier García-Castro, Pilar Sanchez-Gomez, Vicente A. Torres, Verónica Palma

**Affiliations:** a Faculty of Sciences, Laboratory of Stem Cells and Developmental Biology, Universidad de Chile, Santiago, Chile; b Hospital Infantil Universitario Niño Jesús, Madrid, Spain; c Hospital de Pediatría Dr. Prof. Juan P. Garrahan, Buenos Aires, Argentina; d Unidad Anatomía Patológica, Unilabs, Madrid, España; e Faculty of Medicine, Universidad de Chile, Santiago, Chile; f Cellular Biotechnology Unit, ISCIII, Madrid, Spain; g Neurooncology Unit, Chronic Disease Program, ISCIII, Madrid, Spain; h Institute for Research in Dental Sciences and Advanced Center for Chronic Diseases (ACCDiS), Faculty of Dentistry, Universidad de Chile, Santiago, Chile

**Keywords:** Neuroblastoma, Netrin-4, Laminin γ1, Neogenin-1, cell migration, cell adhesion, basal lamina

## Abstract

Neuroblastoma (NB) is the most common pediatric extracranial solid tumor. It arises during development of the sympathetic nervous system. Netrin-4 (NTN4), a laminin-related protein, has been proposed as a key factor to target NB metastasis, although there is controversy about its function. Here, we show that NTN4 is broadly expressed in tumor, stroma and blood vessels of NB patient samples. Furthermore, NTN4 was shown to act as a cell adhesion molecule required for the migration induced by Neogenin-1 (NEO1) in SK-N-SH neuroblastoma cells. Therefore, we propose that NTN4, by forming a ternary complex with Laminin γ1 (LMγ1) and NEO1, acts as an essential extracellular matrix component, which induces the migration of SK-N-SH cells.

## Introduction

Pediatric solid tumors represent about 30% of pediatric cancers. Neuroblastoma (NB) is an extracranial solid tumor that emerges from neural crest cells during development and it is a highly metastatic cancer [1]. The laminin-related secreted Netrins (Netrin 1–4) act as versatile extracellular cues regulating axon guidance [2], angiogenesis, survival and cell proliferation during embryogenesis as well as in cancer [3]. We have recently shown that Netrin-4 (NTN4) promotes NB progression and metastasis acting as a chemotaxis stimulus for the Neogenin-1 (NEO1) receptor [4]. NEO1, a member of the immunoglobulin superfamily of transmembrane protein receptors, and its homologue, the Deleted in Colorectal Cancer receptor (DCC), have been related to tumor progression, proliferation, angiogenesis, apoptosis, and migration in several tissues [5–9]. However, contrasting with our results, NTN4 was recently proposed to signal independently of classical Netrin NEO1/DCC receptors [10]. In line with the former observation, NTN4 protein structure was determined and proposed as a cell adhesion molecule, acting as an extracellular matrix protein that forms a high-affinity complex with laminin γ1 (LMγ1) [11]. Added to this information, data by Staquicini et al. [12] suggested the formation of a complex between NTN4 and LMγ1 that activates a signaling pathway mediated by a6β1 integrin, participating thereby in the migration of neural stem cells. Hence, in this study, we aimed to examine the short range-effects of NTN4 on NB cell migration. To this end, by using the SK-N-SH cell line, we demonstrate a role for NTN4 acting as a cell adhesion molecule in the extracellular matrix, contained within a NTN4/NEO1/LMγ1 ternary complex. Furthermore, our results show that NTN4 is strongly expressed in NB patient samples, in particular in endothelial cells. NTN4 might act both as a cell adhesion and chemotactic stimulus, highlighting the important contribution of the NTN4/NEO1 signaling axis in NB migration and metastasis; a result that might reconcile the apparent controversy in the field and thus provide a new mechanism underlying NB metastasis.

## Results

**Figure 1.**
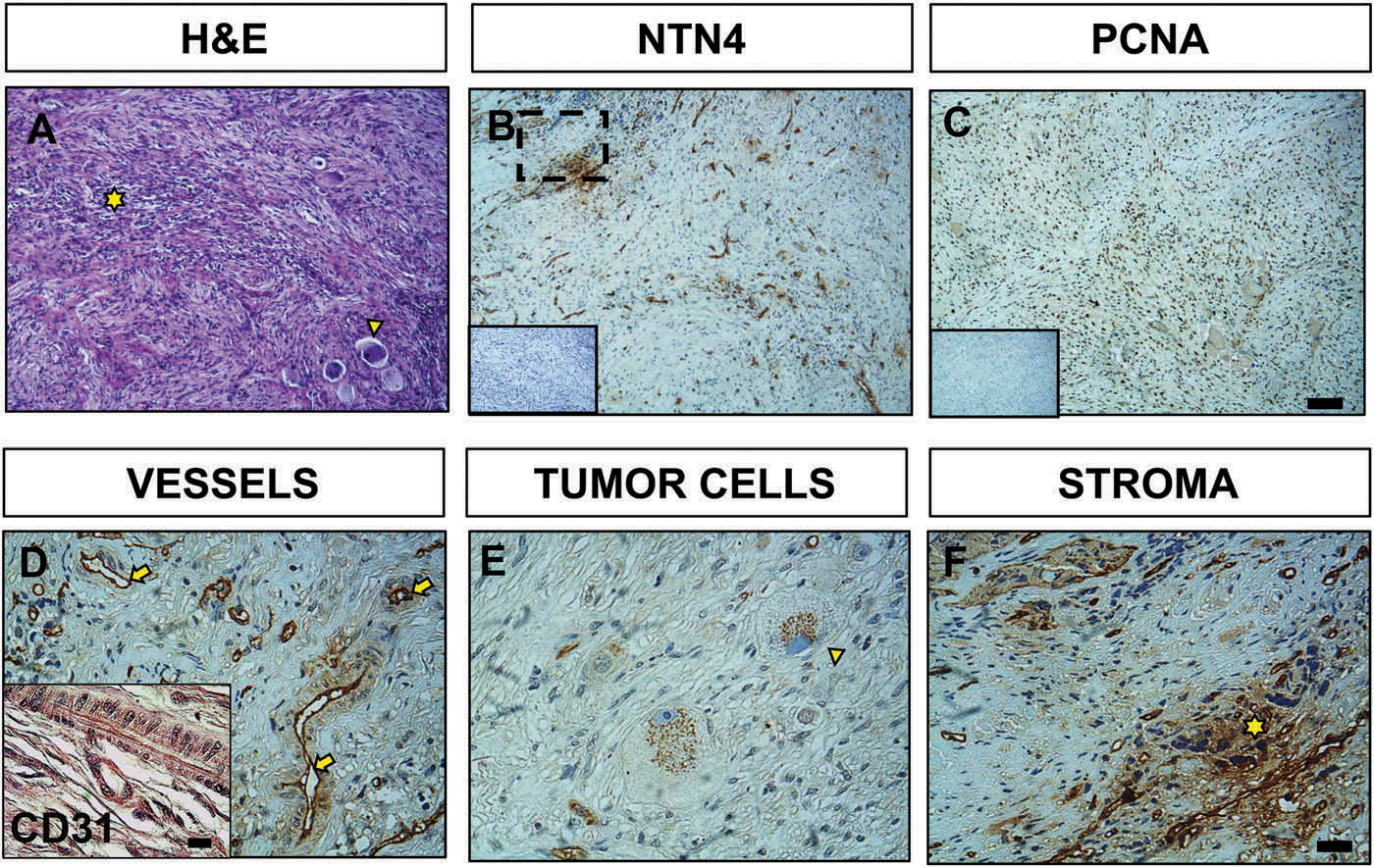
Immunohistochemistry of NTN4 expression within NB. Representative light microscopy images of a neuroblastoma sample from a female patient (age > 18M); tumor with retroperotineal location and in disseminated stage. (a) Hematoxylin and eosin staining reveals presence of ganglionar-differentiated cells (yellow arrowhead) and primitive neuroblasts (yellow asterisk). (b) Immunohistochemistry of NTN4 with its corresponding negative control (inset). (c) PCNA staining. (d) NTN4 is expressed preferentially in the endothelium (yellow arrows), as confirmed by CD31 staining (inset). (e) Detail of ganglionar-differentiated cells with strong NTN4 cytoplasmic expression in a characteristic punctuated pattern (yellow arrowhead). (f) Close up image of (b) as indicated, highlighting NTN4 expression within the stroma (yellow asterisk). A, B, C; Scale bar = 40 μm. D, E, F; Scale bar = 10 μm; inset = 10 μm.

To investigate the role of NTN4 in NB, we first characterized the expression of the ligand in a cohort of 23 NB patient samples. The samples were stratified based on the International Neuroblastoma Risk Group Staging System (INRGSS) which contemplates a pretreatment risk classification system, considering tumor spread and surgical risk factors known as Image Defined Risk Factors (IDRFs) at the moment of diagnosis of the disease. Localized tumors are staged L1 or L2 based on the absence or presence of one or more of 20 IDRFs, respectively. Metastatic tumors are defined as stage M and MS, the latter refers to metastases confined to the skin, liver, and/or bone marrow in children younger than 18 months of age [13]. In order to correlate patient’s disease staging with our NTN4 immunohistochemistry analysis, we organized the results in relation to PCNA expression levels, age, tumor stage, patient status, gender and primary tumor sites. Particularly, we evaluated NTN4 presence in tumor cells, stroma and blood vessels (Table 1). Despite the wide spectrum of NB presentation and clinical course [13–15] our data show that NTN4 is strongly expressed in NB. Notably, the number of male patient’s biopsies expressing NTN4 is twice the number of female biopsies expressing it. Another intriguing result is that no NTN4 expression was found in tumor cells or stroma of patient’s whose primary tumor location was ascribed at the thoracic level. Despite these observations, statistically there is no association between the percentage of NTN4 and these clinical features. Interestingly, NTN4 was intensely expressed in the endothelium (Figure 1) throughout all the samples analyzed, independent of the tumor stage (Figure S1). Corroborating our results, NTN4 indeed has been described as an endothelium lamina basal component in another context such as hemangiogenesis [16]. In a representative NB section, defined by classification criteria as characteristic of a disseminated tumor stage, primitive neuroblasts, identified as small, round and blue cells with almost no cytoplasm, grouped in small nests (Figure 1(a), asterisk) and are easily distinguished from the ganglionar apparent cells. The latter are revealed by hematoxylin and eosin staining as cells with abundant eosinophilic cytoplasm, nucleus with vesicular chromatin and a prominent nucleolus (Figure 1(a), arrowhead). NTN4 expression in this tumor is localized mostly in blood vessels and stroma (Figure 1(b, f)). PCNA expression in this sample is very high (PCNA > 40%), corroborating the aggressive stage of this NB sample (Figure 1(c)). Strong NTN4 expression can be found in the endothelium, seeming to delineate the CD31 positive blood vessels (Figure 1(d), arrows; inset, Figure S2). Within the tumor cells, NTN4 expression is located in the cytoplasm surrounding the nucleus (Figure 1(e), arrowhead). NTN4 expression is also highly upregulated within the extracellular matrix in regions of high cellular density (Figure 1(f), asterisk).

**Table 1.** Characterization of NTN4 expression in patients with NB. Percentage of NTN4 positive samples distinguishing for tumor cells, stroma and/or blood vessel expression, according to specific clinical characteristics of the patients. We do not found association between percentage of NTN4 and clinical features. Asterisk for p value from fisher’s exact test.

Clinical Feature		% of NTN4 positive tumor cells Samples	% of NTN4 positive Stroma Samples	% of NTN4 positive Blood vessel Samples	χ2	df	p
**Gender**	Male	78	78	100	1.23	2	0.54
	Female	36	50	100			
**Age**	> 18M	63	45	100	1.34	2	0.51
	< 18M	42	75	100			
**Tumor Stage**	Disseminated (M, MS)	63	50	100	0.49	2	0.78/0.86*
	Localized (L1, L2)	47	67	100			
**PCNA**	> 40%	45	45	100	0.53	2	0.77
	< 40%	58	75	100			
**Primary Tumor Sites**	Cervical	100	100	100	0.54	8	0.71/0.84*
	Thoracic	0	0	100			
	Abdominal	33	83	100			
	Retroperitoneal	60	60	100			
	Adrenal	60	40	100			
**Patient Status**	Dead	60	40	100	0.43	2	0.81/0.81*
	Recovered	53	67	100

**Figure 2.**
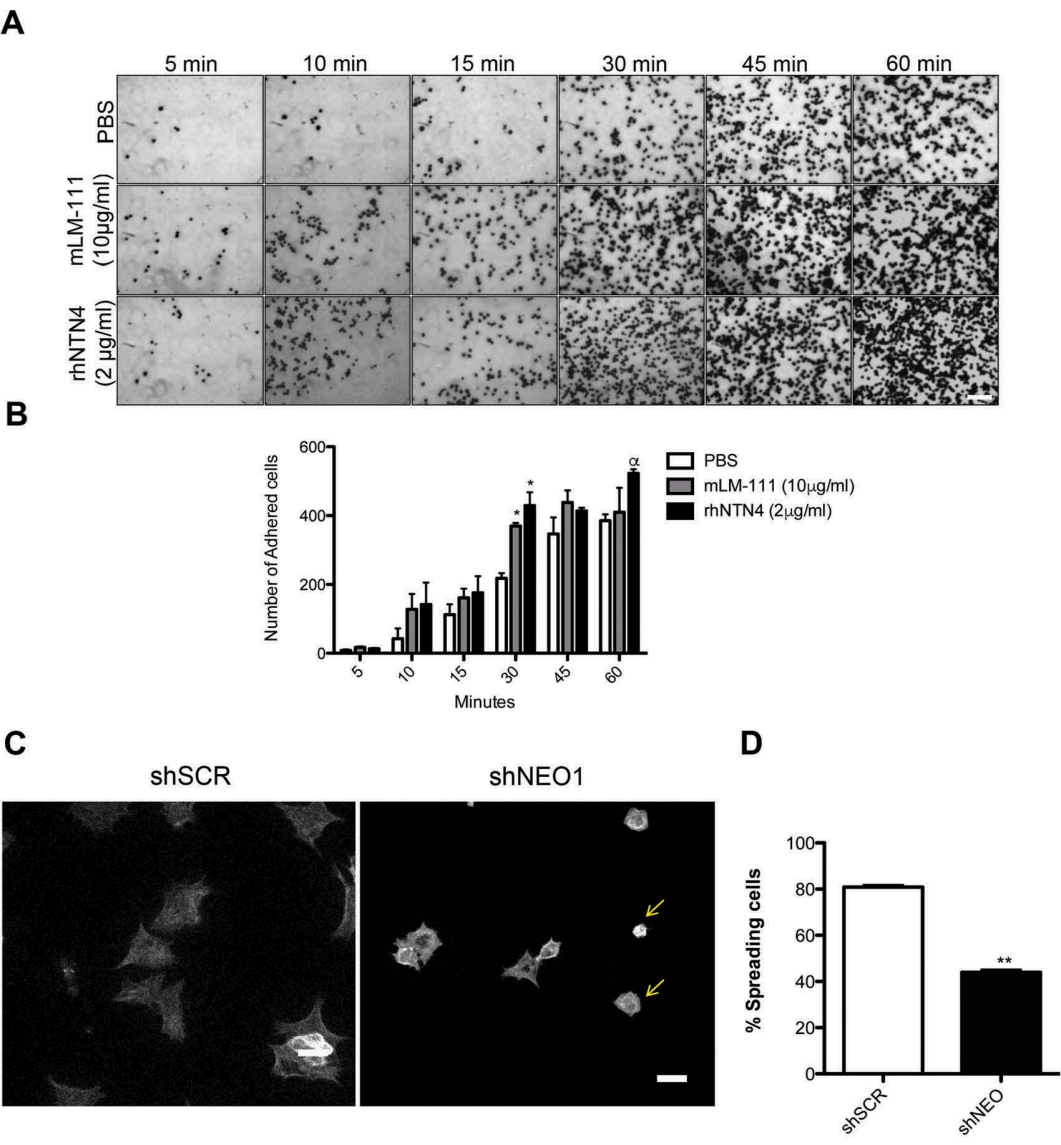
NTN4 as a cell adhesion molecule and NEO1 contributes to cell spreading of SK-N-SH cells. (a) Representative images are shown for cell adhesion assays, using 2 μg/ml rhNTN4 or 10 μg/ml mLM-111 (positive control) at the indicated time points (N = 3). Scale bar = 100 μm. (b) Quantification of cell adhesion assays shown in A. N = 3, n = 15 fields per condition at time points indicated in A. Bonferroni posttest, *p < 0,05 PBS versus mLM-111 or rhNTN4 in 30 min of adhesion, α p < 0,05 PBS versus rhNTN4 in 60 min of adhesion. (c) Representative images of spreading assay performed with shSCR and shNEO1 SK-N-SH cells which spreaded into Fibronectin (2µg/ml) for 1h. Falloidin staining was used to evaluate cell spread. Yellow arrows indicate the different phenotype in spreading of the shNEO1 cells. Bar = 100 µm. (d) Quantification of the spreading assay, where results are expressed as percentage of spreaded cells. n = 23, ** p < 0.01 shSCR versus shNEO1 spreaded cells (black asterisk) or shNEO1 spreaded cells versus no spreaded cells (white asterisk).

**Figure 3.**
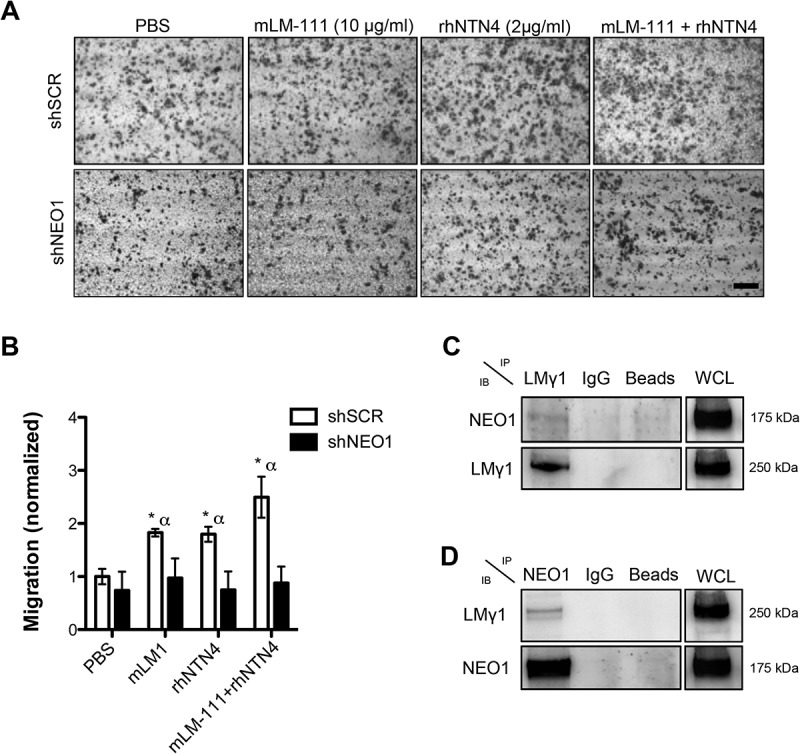
NTN 4 induces cell migration in neuroblastoma cell line, SK-N-SH, through NEO1/Lamimin γ1 interaction (a) Representative images of transwell assays using rhNTN4 and mLM-111 as adhesion molecules in SK-N-SH cells shSCR and shNEO1. Briefly, transwell assays were performed in chambers with an 8μm-pore membrane. Chambers were pre-treated with PBS, 10 μg/ml mLM-111 and/or 2 μg/ml rhNTN4 and placed on the underside of the membrane for 12h before performing the assay. As a chemotactic stimulus DMEM medium supplemented with 5% FBS was used. Cell migration was allowed for 2 h and analysis was performed as described in the material and methods. Data were normalized with respect to shSCR cells (PBS condition). Scale bar 100 μm. (b) Quantification of transwell assays obtained in C. Migrated cells were counted using inverted microscopy at 100x magnification. Five fields per condition were counted and data represent the average from three independent experiments. Data was normalized to shSCR cells (PBS condition). Mann Whitney t-test *p < 0,05 shSCR PBS versus shSCR mLM-111, rhNTN4 or both, α p < 0,05 shSCR versus shNEO1 in the same cell adhesion stimulus. (c), (d) Representative western blots of protein co-immunoprecipitation assays used to evaluate interaction between NEO1 and LMγ1 in SK-N-SH cells. Cells were lysed and incubated using specific antibodies against either LMγ1 (c) or NEO1 (d) followed by western blot against NEO1 and LMγ1.

Next, we aimed to examine the effects of NTN4 on NEO1 driven cell adhesion and migration. To this end, we evaluated first whether NTN4 behaves as an adhesion molecule in a NB cell line. We performed an adhesion assay with SK-N-SH cells, at different times, using rhNTN4 (2 μg/ml, according to [11]), or mouse Laminin-1 (10 μg/ml) (mLM-111), as a positive control. As indicated in Figure 2(a), NTN4 acts as an adhesion molecule, as corroborated by quantification (Figure 2(b)), revealing significant differences at 30 min of adhesion, when compared with the PBS control condition. Importantly, NTN4 acted as an adhesion molecule, inducing the adhesion of SK-N-SH cells to a similar extent as compared with mLM-111. In order to determine whether NEO1 is also important to adhesion in SK-N-SH cells, we performed a spreading assay using control cells (shSCR) or NEO1 knockdown cells (shNEO1), (Figure 2(c)), demonstrating that shNEO1 cells spread less than control cells on a Fibronectin substrate, indicating that NEO1 indeed contributes to cell adhesion and spreading (Figure 2(d)). Furthermore, to evaluate the requirement of NEO1 in the context of a cell adhesion induced-migration, provided particularly by NTN4, a transwell assay was tested in shSCR or shNEO1 cells. rhNTN4 and/or mLM-111 were placed at the transwell and the assay was performed according to [17] using low serum as a chemotactic stimulus in the bottom part of the chamber. As shown in Figure 3(a) shNEO1 cells migrated less than the shSCR cells in both conditions, mLM-111 and rhNTN4, even when using a combination of both molecules. Quantifications revealed no significant differences between shSCR and shNEO1 cells at basal migration (i.e., using PBS as stimulus, Figure 3(b)), probably due to dispersion of the data. However, when using either mLM-111 or rhNTN4, migration was 2-fold increase in shSCR, with respect to shNEO1 cells, further supporting that NTN4 promotes cell adhesion and migration to a similar extent as for mLM-111 . According to the observations of Reuten et al. [11], cell migration is likely modulated by NEO1, since silencing of the receptor prevented cell migration. Finally, combined use of mLM-111 and NTN4 led to a non–significant increase in cell migration of shSCR cells, when compared to each separate ligand. In our previous work [4], NTN4 was postulated as a chemotactic molecule that promotes migration of NB cells. Now, based on these observations, we reasoned that NTN4 could also act as an adhesion molecule, in conjunction with Laminin LMγ1.

According to Reuten et al. [11], NTN4 does not interact directly with any of the putative Netrin´s family canonical receptors, such as NEO1. However, others and we have shown that NTN4 immunoprecipitates with NEO1 [4,7]. This suggests that the interaction between NEO1 and NTN4 is rather indirect, most probably forming part of a complex with LMγ1. Indeed, studies by Staquinini et al. [12] and Reuten et al. [11] demonstrated an interaction between NTN4 and LMγ1. To assess whether NEO1 also combines with LMγ1 and thus could explain a functional protein complex linking NTN4 with NEO1, we performed co-immunoprecipitation assays. Reciprocal co-immunoprecipitation experiments demonstrated that such association is effective on SK-N-SH cells (Figure 3(c, d)). These results show that NEO1 associates with the LMγ1 chain and presumably, this interaction accounts for the reduction of cell migration observed in NEO1 knockdown cells.

Taken together, our results confirm that NTN4 acts as a cell adhesion molecule, promoting serum-induced cell migration in the NB cell line SK-N-SH through a ternary complex formed by NTN4/NEO1/LMγ1.

## Discussion

The spectrum of NB and prognosis is wide, ranging from an aggressive course with poor survival, differentiation of NB into more differentiated ganglio-neurona, or even spontaneous tumor regression. Due to the high metastatic rate of NB targeted therapies, aimed at modulating those critical processes that are critical for tumor growth and metastasis, are required.

NTN4 is a protein involved in many physiological processes, such as angiogenesis [7], neovascularization [18], axon branching [19] among others. NTN4 has been implicated as a prognosis marker of certain malignancies such as gastric [20] and breast cancer [21]. In both cancers, it has been shown that NTN4 has a role in the migration and/or metastasis of tumor cells, without specifying the mechanism associated with these functions. Based on our results, we can state that NTN4 is expressed in all NB samples, although with different cellular distribution. By analyzing the patient cohort samples, NTN4 reveals labeling in the endothelium of all sections (Figure S2), probably delineating the basal lamina [22]. In agreement with our observations, NTN4 has been described as integral component of the basal lamina of the endothelium and is highly enriched in the proximal basement membrane of tubules [23]. Indeed, our studies revealed positive LMγ1 staining in blood vessels located within the tumor niche (Figure S2). Moreover, our analysis suggests that NEO1 can also be expressed in blood vessels (Figure S2). Probably, tumor cells are attracted to endothelial capillaries as they disseminate and, NTN4, produced as a chemotactic molecule in this permissive substrate, might facilitate their migration. NTN4 is also observed in the stroma, especially in samples that have the histological appearance of neuropil. In tumor cells, NTN4 labels in a distinctive punctuated pattern, suggestive of strong expression in secretory organelles, observations that need to be further defined. Thus, NTN4 could be acting both in autocrine and paracrine fashion in the tumor niche.

Interestingly, the expression of NTN4 in primary tumors located in the thoracic region was null. Supplementary studies are required aiming to increase the number of samples with this location of NB in order to be able to assure that there is a relationship between the location of the primary tumor and the absence of NTN4.

Historically, NTN4 has been postulated as a chemotactic molecule [4,19]. Nevertheless, a recent study demonstrated that this molecule could be a putative component of the extracellular matrix, through its high affinity with LMγ1, evidencing that secreted NTN4 could function in an autocrine fashion [11]. Our group has shown that NTN4 promotes the migration, survival, and metastasis of NB cells through NEO1 by acting as a chemotactic molecule on NB cells [4]. Here, we show that NTN4 also could act as a cell adhesion molecule inducing cell migration. In addition, we provide evidence that NEO1 contributes to cell spreading, indicating its function in the initiation of cell migration. Transwell migration assays adding rhNTN4 in the underside of the chamber generated a positive cell migration of control NB cells (shSCR), similar to the results obtained with mLM-111 . Interestingly, when using a combination of mLM-111 and rhNTN4, cell migration was not significantly different to that observed using separate ligands, although there is a tendency. Knockdown of NEO1 reduces the cell migration of SK-N-SH cells in all conditions, except in the non-stimulated control (PBS), revealing that NEO1 is also indispensable for adhesion-induced migration. This phenomenon could be explained on the basis that NEO1 associates with LMγ1 in this context. It is known that LMγ1 binds to integrin α6β1 [24]. Hence, most probably the interaction of NEO1 with NTN4 shown in [4], could be explained due to the formation of a ternary complex, including NTN4, LMγ1 and NEO1. At this point, we do not rule out that integrins also form part of the complex, a matter that deserves further investigation. Importantly, the combined action of all these molecules may be a key signaling event driving NB migration and dispersion.

## Materials and methods

### Patient samples

Ethics committees from University of Chile and CONICYT approved this study. General written consent was obtained from all the patients enrolled by HNPG, at diagnosis. All human tumor samples used in this study were diagnosed, and morphologically typified, through histological analysis at the Anatomopathology Center of this institution.

### Immunohistochemistry and histological analysis

Paraffin-embedded samples of NB were deparaffinated and rehydrated as in [25]. Immunohistochemical assays were proceeded incubating the tissues with primary antibodies anti-human NTN4 (AF1254, goat, R&D Systems), anti LMγ1 (MAB1920, Millipore), anti CD31 (P8590, mouse, Sigma), anti NEO1 (H-175, Santa Cruz) and anti-human PCNA (13–3900, mouse, Invitrogen), overnight at 4°C and subsequently with the secondary biotinylated anti-goat Igg (R&D Systems) for NTN4 analysis, and biotinylated anti-rabbit/mouse IgG (Vector Labs) for PCNA analysis for one hour at room temperature (25°C). The samples were later revealed with 39-diaminobenzidine (DAB, Roche). Samples were stained with Hematoxilin (Vector Laboratories, Burlingame CA) and Eosin Y (Sigma Aldrich, St Louis, MO). Immunofluorescence samples were incubated an extra hour with a Donkey anti goat Alexa Fluor 555 (Invitrogen). Dapi was used for nuclei staining. Slices were mounted with fluorescence mounting medium (Dako). PCNA percentage was calculated by counting by two independent observers the number of cells marked in quadrants and multiplying by the total number of quadrants present in each sample. 40% was the median obtained for the total of 23 samples. χ square and Fisher’s exact test (n < 5 samples) were as statistical tests.

### Cell culture

The NB cell line SK-N-SH, was cultured in high glucose Dulbecco’s Modified Eagle Medium (DMEM, Invitrogen) with 5% fetal bovine serum (FBS, Gibco) and supplemented with antibiotics (Penicillin-Streptomycin, 10,000 U/mL). To knock-down NEO1 (shNEO1), SK-N-SH were transduced according to [4] assessing knockdown efficiency via Western Blot. Stable shNEO1 and shSCR (control) SK-N-SH cells were previously established, using puromycin as a selection marker, as indicated in [4].

### Cell adhesion assay

48-well plates were incubated overnight at 4ºC with PBS, mouse Laminin-1 (mLM-111) (Invitrogen, extracted from Engelbreth-Holm-Swarm sarcoma) (10μg/ml) or rhNTN4 (2μg/ml). Next, SK-N-SH cells (70,000) were placed into the wells and allowed to adhere at different times (0, 5, 10, 15, 30, 45 and 60 minutes). At indicated time points, cells were fixed and stained using 0.1% crystal violet in 20% methanol saline (0.15 M NaCl). Photographs were taken to evaluate adhesion and results are presented as the number of adherent cells per condition.

### Spreading assay

ShSCR and shNEO1 SK-N-SH cells were seeded on coverslips pre-covered with Fibronectin (2 μg/ml) for 1 h. Then, the cells were fixed with PFA 4% w/v followed by staining with phalloidin-546 (Thermofisher) and DAPI. Cells were observed and documented by confocal microscopy (Zeiss 710). A total of 23 spreading cells was quantified per condition.

### Transwell migration assays

Transwell assays were completed using a chamber within an 8μm-pore polycarbonate membrane (Corning). As a cell adhesion stimulus, 10 μg/ml mLM-111 (Invitrogen) and/or 2 μg/ml rhNTN4 (R&D systems) were placed on the underside of the transwell membrane 12h before performing the assay, dissolved in Phosphate buffered saline (PBS), which was used as a control. As a chemotactic stimulus DMEM 5% Fetal Bovine Serum (FBS) was used at the bottom of the chamber. Briefly, 50,000 shNEO1 or shSCR SK-N-SH cells were placed in the upper chamber. The cells were incubated for 2h, fixed and stained with crystal violet solution. All results were normalized with respect to the PBS condition of the shSCR cells.

### Protein co-immunoprecipitation

SK-N-SH cells were used to prepare cell extracts with a buffer containing 20 mM Tris, pH 7.4, 150 mM NaCl, 1% NP-40, and protease inhibitors by 5 min incubation on ice. Samples were centrifuged at 13,000 × *g* by 1 min at 4°C, and supernatants (1000 μg total protein) were immunoprecipitated with Dynabeads protein A (Thermofisher) bead-immobilized antibodies for 1h. NEO1 was immunoprecipitated with 2 μg of a rabbit polyclonal antibody (H-175, Santa Cruz) and LMγ1 was immunoprecipitated with 2 μg of mouse monoclonal antibody (MAB1920, Millipore). Immunoprecipitated samples were solubilized in loading buffer with ß-mercaptoethanol, and analyzed by Western blot as indicated in (Figure 3).
