# Supplementary material for: The Netrin-4/Laminin γ1/Neogenin-1 complex mediates migration in SK-N-SH neuroblastoma cells
Source: Cell Adh Migr. 2018 Aug 30;13(1):33–40. doi: 10.1080/19336918.2018.1506652 (PMC6527380; doi:10.1080/19336918.2018.1506652)
Supplement: Supplemental Material [file kcam-13-01-1506652-s001.zip › Supplemental figure 1&2 II resubmission (1).docx]

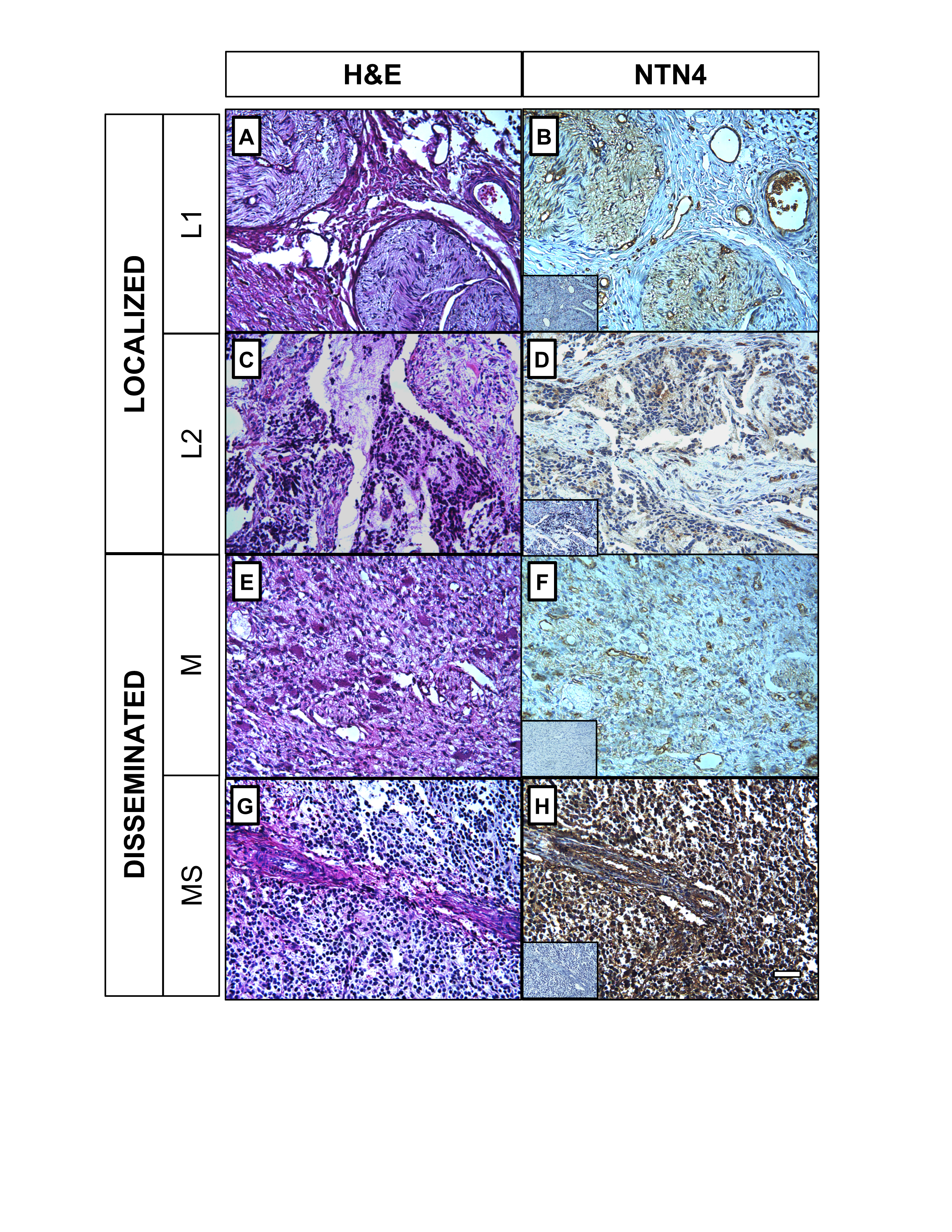


Supplemental figure 1:

**NTN4 expression within NB tumors** **stratified based on the International Neuroblastoma Risk Group Staging System (INRGSS) .**

(A), (C), (E), (G) Hematoxylin and eosin staining of NB sections at stages as indicated. (B), (D), (F), (H) Analysis of NTN4 expression within the corresponding NB sample. Each immunohistochemistry is shown with its corresponding negative control (inset). Scale bar = 10 μm.


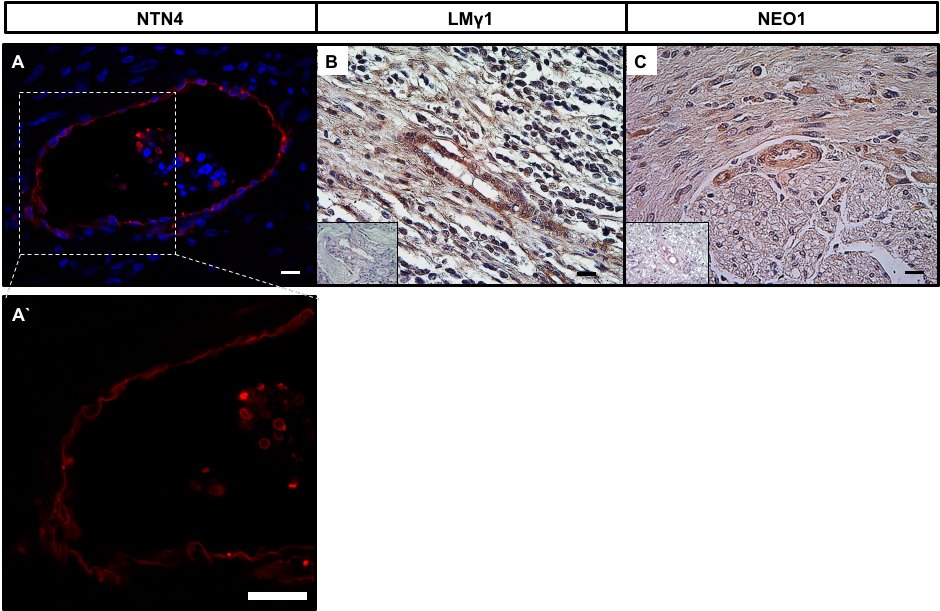


Supplemental figure 2:

**NTN4, LMγ1 and NEO1 expression in blood vessels within NB tumors.**

(A, A’) Representative images of NTN4 staining (red) in blood vessels of the same patient shown in figure1. DAPI (blue) was used for nuclei staining. (B) LMγ1 immunostaining identifies basement membrane. (C) NEO1 immunohistochemistry shows localization on blood vessels. Negative controls are shown as insets. Scale bar = 10 μm.
